# Alcohol Reward Is Increased after Roux-en-Y Gastric Bypass in Dietary Obese Rats with Differential Effects following Ghrelin Antagonism

**DOI:** 10.1371/journal.pone.0049121

**Published:** 2012-11-07

**Authors:** Andras Hajnal, Alevtina Zharikov, James E. Polston, Maxine R. Fields, Jonathan Tomasko, Ann M. Rogers, Nora D. Volkow, Panayotis K. Thanos

**Affiliations:** 1 Department of Neural and Behavioral Sciences, The Pennsylvania State University College of Medicine, Hershey, Pennsylvania, United States of America; 2 Department of Surgery, The Pennsylvania State University College of Medicine, Hershey, Pennsylvania, United States of America; 3 Laboratory of Neuroimaging, National Institute on Alcohol Abuse and Alcoholism Intramural Program, National Institutes of Health, Bethesda, Maryland, United States of America; 4 Behavioral Neuropharmacology and Neuroimaging Lab, Brookhaven National Laboratory, Upton, New York, United States of America; St. Joseph's Hospital and Medical Center, United States of America

## Abstract

Roux-en-Y gastric bypass (RYGB) is one of the most successful treatments for severe obesity and associated comorbidities. One potential adverse outcome, however, is increased risk for alcohol use. As such, we tested whether RYGB alters motivation to self-administer alcohol in outbred dietary obese rats, and investigated the involvement of the ghrelin system as a potential underlying mechanism. High fat (60%kcal from fat) diet-induced obese, non-diabetic male Sprague Dawley rats underwent RYGB (n = 9) or sham operation (Sham, n = 9) and were tested 4 months after surgery on a progressive ratio-10 (PR10) schedule of reinforcement operant task for 2, 4, and 8% ethanol. In addition, the effects of the ghrelin-1a-receptor antagonist D-[Lys3]-GHRP-6 (50, 100 nmol/kg, IP) were tested on PR10 responding for 4% ethanol. Compared to Sham, RYGB rats made significantly more active spout responses to earn reward, more consummatory licks on the ethanol spout, and achieved higher breakpoints. Pretreatment with a single peripheral injection of D-[Lys3]-GHRP-6 at either dose was ineffective in altering appetitive or consummatory responses to 4% ethanol in the Sham group. In contrast, RYGB rats demonstrated reduced operant performance to earn alcohol reward on the test day and reduced consummatory responses for two subsequent days following the drug. Sensitivity to threshold doses of D-[LYS3]-GHRP-6 suggests that an augmented ghrelin system may contribute to increased alcohol reward in RYGB. Further research is warranted to confirm applicability of these findings to humans and to explore ghrelin-receptor targets for treatment of alcohol-related disorders in RYGB patients.

## Introduction

The epidemic of obesity and its associated health consequences represent a major cause of preventable death. At present, Roux-en-Y gastric bypass (RYGB) surgery is the most effective method to achieve significant, long-term weight loss [1]. It is estimated that over 200,000 procedures were performed in the United States in 2009 [2]. Following RYGB, patients typically lose approximately 30% of total body weight or 60–70% of excess body weight [3]. Although the exact mechanism remains unknown, it is believed that factors other than restriction and malabsorbtion of the ingested food may contribute to the beneficial effects of RYGB surgery [4], [5]. Following RYGB, patients voluntarily restrict consumption of calorie-dense, highly palatable foods such as fats, concentrated carbohydrates, ice cream, and sweetened beverages [6], [7], [8], [9]. Such behavioral changes seem to be independent of perioperative counseling of the patients, as they also occur in animal models of RYGB.

Specifically, recent reports have demonstrated reduced preferences and motivation for sugars and fats following RYGB in normal weight or obese rats [8], [10], [11], [12], [13]. Thus, it appears that RYGB may reduce hedonic (palatability) and/or incentive (rewarding) effects elicited by certain foods. Conversely, concerns have been raised by clinical reports of an increased risk for ethanol (EtOH) consumption following RYGB surgery [14], [15], [16], [17]. Due to these concerns, ethanol abuse represents a relative contraindication for surgery in most bariatric surgery programs [18].

Currently, discrepancies exist in the literature with respect to actual consumption of alcohol following surgery. Several investigations indicate there is increased risk for alcohol following RYGB surgery [14], [15], [16], [17], [19]. However, other studies show no change in risk for alcohol following RYGB [20], [21]. However, the broader consensus is that RYGB patients have higher and longer-lasting blood alcohol concentrations, and a shorter period of onset than non-surgical controls when consuming similar amounts of ethanol [22], [23], [24], [25]. Changes in alcohol's pharmacokinetics may alter not only the bioavailability and stimulating properties of EtOH acting directly on the brain, but may also influence the neuronal and hormonal signals upstream of the reward system. To our best knowledge, there are only two animal studies that have investigated alcohol intake in a rat model of RYGB. One report indicates that there is actually decreased risk for alcohol abuse following RYGB surgery in ethanol preferring rats [26]. In contrast, using a two-bottle choice paradigm in outbred high fat diet-induced obese rats we found that RYGB rats preferred lower concentrations of alcohol (2 and 4%) and consumed twice as much as sham-operated obese controls and 50% more than normal-diet lean controls [27].

**Figure 1:**
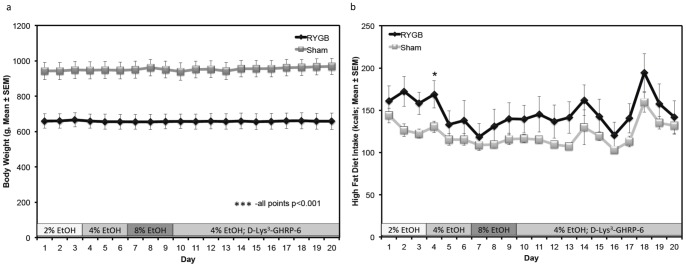
RYGB and Sham rats differed significantly in body weight after bypass or sham surgeries, but not high fat diet intake. a) Daily body weights (in grams) of RYGB and Sham rats beginning 4 weeks after the Roux-en-Y gastric bypass or Sham surgical procedures were averaged and are presented as Mean ± SEM. Throughout the alcohol testing period, RYGB rats (black diamonds) had significantly lower body weights on each day of testing. *** p<0.001, all days. b) Daily intake of high fat diet (in grams) by RYGB and Sham rats throughout the alcohol testing period is presented as Mean ± SEM. Throughout the majority of the testing period there was no significant difference in intake; RYGB rats (black diamonds). * p<0.05.

Of special relevance, recent imaging studies have revealed changes in RYGB patients dopamine D2 receptor (D2R) expression in the ventral striatum and caudate nucleus [28], [29], an area involved with alcohol's rewarding effects [30] and also associated with susceptibility for alcohol use and abuse [31], [32], [33], [34]. Furthermore, hormones that have been shown to change after RYGB, such as leptin and ghrelin [35], [36], [37], are also known to modulate the dopaminergic reward system [38], [39], [40], [41] as well as alcohol consumption [41], [42], [43]. Therefore, it is quite surprising that the extent to which RYGB may alter the motivation to consume alcohol has only been investigated in two studies none of which assessed either incentive or consummatory aspects of reward directly [26], [27]. The study by Davis et al. compared self-reported alcohol consumption before and after bariatric surgery in moderate drinkers and used conditioned place preference (CPP) as a measure of alcohol reinforcement in a selectively-bred alcohol preferring rat strain. Our previous study [27] used normal outbred dietary obese Sprague Dawley rats, which would be more representative of the RYGB patient population not expressing increased susceptibility to alcohol use, but our study only investigated the 2-bottle choice-preferences between alcohol and water.

**Figure 2:**
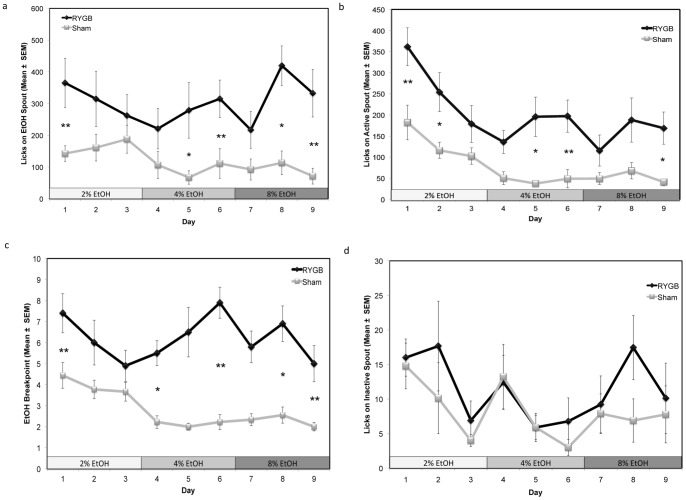
RYGB rats worked harder for, and consumed more alcohol during oral self-administration. a) EtOH intake, measured by the number of licks made on the spout containing ethanol during PR testing, was averaged and presented as Mean ± SEM. RYGB rats made more licks for 2% (Day 1), 4% (Days 5–6), and 8% EtOH(Days 8–9). b) Active spout licks, presented as Mean ± SEM. RYGB rats made more licks on the active spout than Sham rats for 2% (Days 1–2), 4% (Days 5–6), and 8% EtOH (Day 9). c) The number of cycles completed by the rats (breakpoint) presented as Mean ± SEM. RYGB rats displayed significantly higher breakpoints than Sham rats for 2% (Day 1), 4% (Days 4,6), and 8% EtOH (Days 8–9). d) Inactive spout licks, presented as Mean ± SEM. There were no significant differences in the number of inactive licks made between RYGB and Sham rats. * p<0.05; ** p<0.01.

Accordingly, the present study utilized an operant self-administration task to directly evaluate the incentive motivation and drug reinforcement of orally self-administered alcohol after RYGB or Sham operations. Furthermore, a progressive ratio schedule of reinforcement operant licking paradigm originally developed by Sclafani and Ackroff [44] was used, as it has been utilized successfully in our laboratory to measure the reinforcing value of sucrose solutions [45]. Progressive ratio schedules offer information concerning the degree to which a pharmacological agent is reinforcing by defining a breakpoint, or the number of operant responses at which the subject ceases to engage in drug-seeking behavior. In other words, the breakpoint is a measure of how willing the animal is to work for the reward. Therefore, in the present study we measured both operant responses for reward and lickometer responses for EtOH intake, which we believe captures both the appetitive and consummatory aspects of motivation, respectively.

**Figure 3:**
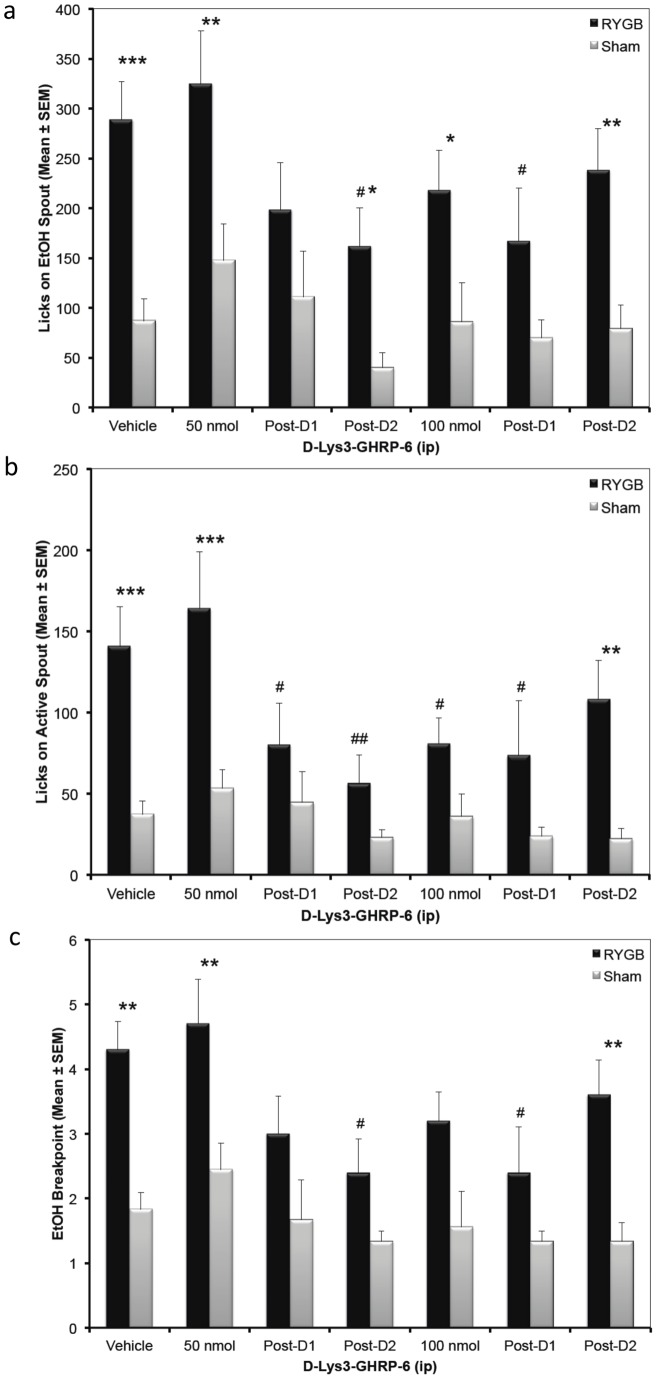
Antagonism of ghrelin receptors resulted in decreased reward and delayed reductions of EtOH-motivated behaviors with an overall increased sensitivity in RYGB rats. a) EtOH intake, measured by the number of licks made on the spout containing ethanol during PR testing was measured following peripheral (IP) injection of the ghrelin receptor antagonist D-[Lys3]-GHRP-6 (GHRP-6) and presented as Mean ± SEM. A delayed within group effect was observed in RYGB rats 2 days after testing with 50 nmol D-[Lys3]-GHRP-6, and 1 day after testing with 100 nmol D-[Lys3]-GHRP-6, while RYGB and Sham rats showed significant between group differences during several test sessions. b) Active spout licks, measured following peripheral (IP) injection of the ghrelin receptor antagonist D-[Lys3]-GHRP-6 (GHRP-6) and presented as Mean ± SEM. RYGB rats showed significant reductions immediately following 100 nmol D-[Lys3]-GHRP-6 and made significantly fewer licks on the days following both doses. c) The number of cycles completed by the rats (breakpoint), measured following peripheral (IP) injection of the ghrelin receptor antagonist D-[Lys3]-GHRP-6 (GHRP-6) and presented as Mean ± SEM. RYGB rats demonstrated reduced breakpoints on subsequent days following D-[Lys3]-GHRP-6 at both doses. * p<0.05; ** p<0.01, *** p<0.001 RYGB compared to Sham. # p<0.05, ## p<0.01 within group comparison vs. vehicle.

An additional aim of the study was to test the potential involvement of ghrelin in increased alcohol self-administration following RYGB. Ghrelin, a hormone produced mainly by the stomach, was first identified as endogenous ligand for the cloned growth hormone [46] and has been shown to increase food intake in both rodents [47] and humans [48]. In human, however, there is an inverse relationship between circulating ghrelin levels and fat mass [49] suggesting that obesity is not caused by hyperghrelinaemia. Recent literature shows ghrelin increases ethanol consumption and ghrelin 1A receptor (GHS-R1A) antagonists block the rewarding effects of alcohol in rodents [43], [50]. Most studies find changes in plasma ghrelin concentrations after RYGB (see, [35] for a review), either in the fasted state or following a meal [36], [51], [52]. Previous reports have shown that ghrelin resistance can occur following diet induced obesity in rodents [53]. This effect may be reversed by RYGB, perhaps resulting in increased responsiveness to ghrelin effects following the surgery. We hypothesized that if enhanced sensitivity to the rewarding effects of alcohol following RYGB is related to improved ghrelin signaling on GHS-R1A, RYGB and Sham-operated controls would display differential sensitivity to the effects of blockade of endogenous ghrelin receptor activation with the selective GHS-R1A antagonist D-[Lys3]-GHRP-6 during alcohol self-administration sessions.

**Table 1:** Inactive licks were not altered significantly by ghrelin antagonism.

D-[Lys3]-GHRP-6 or Vehicle (ip) Inactive Licks (Mean ± SEM)							
	Vehicle	50 nmol	Post- D1	Post- D2	100 nmol	Post- D1	Post- D2
**RYGB**	6.9± 1.7	7.9± 2.2	4.4± 2.2	5.5± 2.6	9.7± 3.5	7.6± 3.0	11.9± 8.4
**Sham**	2.6± 0.7	6.9± 2.5	5.7± 2.5	3.2± 1.9	4.3± 1.4	4.1± 1.9	8.4± 4.4

Injection of the Ghrelin receptor antagonist D-[Lys3]-GHRP-6 did not produce significant changes in the number of licks made on the inactive spout, indicating that the effects were not due to a general reduction in activity.

## Materials and Methods

### Ethics Statement

All experiments were carried out in strict accordance with the recommendations in the Guide for the Care and Use of Laboratory Animals of the National Institutes of Health and Pennsylvania State University College of Medicine Institutional Animal Care and Use Committee guidelines and approved protocols.

### Subjects

A total of 25 (4 weeks of age) male Sprague-Dawley rats (Charles River, Wilmington, MA) weighing 250–275 g at the beginning of the study were used. Rats were housed in individual cages and maintained on a 12∶12-hr light-dark cycle (lights on at 0700) in a temperature and humidity-controlled vivarium. Maintenance diet and water was available ad libitum throughout the experiment except prior to oral glucose tolerance test (OGTT) when food was removed overnight (∼16 hrs) and during the operant test sessions when food and water were removed (for 2 hrs in the morning). Additionally, the rats were put on a water-restriction regimen limiting water access to 2 hrs in the afternoon during an initial habituation period (4 days) to the self-administration continuous access licking task.

### Diet

All rats included in the study were maintained on a nutritionally complete high fat diet (#D12492; Research Diets, New Brunswick, NJ) consisting of 5.24 kcal/gram (60% kcal from fat, 20% kcal from carbohydrates and 20% kcal from protein). High fat diet feeding began at 5 weeks of age, and animals were maintained on this diet for at least 26 weeks prior to experiments and throughout the whole study.

### Body Weight and Food Intake

Body weight and food intake were measured weekly except during the perioperative period (3 weeks) and the EtOH testing period (3 weeks) when measurements were taken daily.

### Drugs

Sucrose (Fisher Scientific, Fair Lawn, NJ) used for self-administration operant training was diluted in deionized water to 10% w/v and prepared daily. Ethanol (95%, Pharmco Products Inc., CT) was diluted in deionized water to 2, 4, 8% v/v and prepared weekly. The selective GHS-R1A antagonist D-[Lys3]-GHRP-6 (His-D-Trp-D-Lys-Trp-D-Phe-Lys-NH2; Tocris Bioscience, Ellisville, MO) was dissolved in sterile 0.9% saline and administered intraperitoneally (IP) at doses of 50 nmol/kg and 100 nmol/kg, 30 min prior to the Progressive Ratio-10 (PR10) sessions.

### Roux-en-Y Gastric Bypass Surgery

Animals were maintained on the high fat diet for 26–28 weeks prior to receiving either RYGB or Sham operation. A detailed description of the surgical technique and perioperative care for RYGB has been published, except that Sprague-Dawley rats were used in the present investigation [10]. Briefly, rats were fasted overnight, but allowed ad-lib water prior to the surgery. Anesthetized rats (isofluorane: 3% for induction, 1.5% for maintenance) were maintained under sterile conditions and were pretreated with a prophylactic antibiotic (Ceftriaxone: 100 mg/kg, im; Roche, Nutley, NJ). An abdominal incision was made on the midline and, in the RYGB surgeries, the stomach was divided to create a reduced (20%) gastric pouch using a blue load GIA stapler (ETS-Flex Ethicon Endo surgery, 45 mm). Measuring from the ligament of Trietz, the small intestine was divided to create a 15 cm biliopancreatic limb and a 15 cm alimentary “Roux” limb, with the remaining segment (65–70 cm) forming the common channel. The gastrojejunal and jejunojejunostomies were performed end-to-side using interrupted 5–0 polypropylene sutures and the abdominal wall and skin were closed using 3–0 silk and 5–0 nylon. The Sham operated controls received manipulation of the stomach and a transverse enterotomy at the same position of the proximal jejunum; however, this was reclosed with interrupted 5–0 polypropylene sutures without forming an anastomosis. The stomach was freed from caudal attachments, and a space along the lesser curve was cleared of attachments as if a stapler was to be inserted. However, the stomach was then replaced to its anatomic location without further manipulation.

All surgical incisions were treated with 0.5 ml of subcutaneous 0.25% bupivicaine to minimize postoperative pain. Further postoperative care included treatment with normal saline (sc; 50 ml/kg, immediately prior to surgery and after surgery, and on postoperative day 1) and buprenorphine (0.5 mg/kg, im) as needed for pain. Animals were given 24 hrs for the anastomoses to heal before being allowed to eat or drink, at which point animals were maintained on a liquid diet consisting of BOOST® (Nestle Nutrition, Minneapolis, MN) and ad lib water for 3 days. On postoperative day 3, the Boost was removed and animals were returned to their high fat diet.

### Oral Glucose Tolerance Test

To test if impaired glucose control (i.e. due to high fat diet and/or obesity) was present and could potentially contribute to behavioral differences observed between surgical groups, an oral glucose tolerance test (OGTT) was performed 3 weeks before the beginning of the alcohol tests (∼3 months postoperatively, and ∼40 weeks on the diet). For the test, the rats were fasted overnight for a minimum of 16 hours and then given glucose (1.25 g/kg, 500 g/l) by intragastric gavage. Blood was taken from nicks made in the tail without anesthesia at 0, 30, 60, and 120 minutes after glucose administration. Blood glucose levels were determined using a glucometer (OneTouch, LifeScan Inc., Milpitas, CA). Animals were classified as diabetic if the peak plasma glucose level was above 16.8 mmol/l (300 mg/dl) at any time point after glucose administration or a glucose level of 11.2 mmol/l (200 mg/dl) at 120 min or later following glucose administration.

### Apparatus

Testing took place in one of six identical operant chambers (MED Associates, St. Albans, VT) in a testing environment separate from the colony room starting about 4 months after the surgeries (i.e. about 10 months on the high fat diet). All chambers had clear Plexiglas tops, front, and back walls. The chambers measured 30.5×24.0×29.0 cm (length x width x height) with a grid floor above a removable waste tray. Each chamber was equipped with three retractable sipper spouts that entered through 1.3 cm diameter holes placed 16.4 cm apart. Each chamber was equipped with a 25W house light within a light and sound attenuated cubicle fitted with a white noise source (75 dB). Ethanol reinforcement was monitored by a lickometer circuit in which licks on an empty bottle (the active or operant spout) triggered deployment of a second bottle containing 2, 4, or 8% EtOH for 10 seconds, during which time licks from the EtOH spout were recorded.

### Training, PR, and Drug Schedule

Our standard progressive ratio (PR) training and testing procedures have been described in detail [54]. Briefly, rats were overnight water deprived for continuous access training. For four days, rats received 30 min access to water in the operant chambers, with an additional 2 hrs of access to water each afternoon in their home cages to ensure proper hydration. Following the 4 days of water training, rats were returned to *ad libitum* water access, except that during the testing period subjects were water and food deprived for 2 hr each morning. Sucrose training then commenced, with rats given six consecutive days of training using 10% sucrose on a PR10 schedule of reinforcement to ensure thorough shaping of the operant response. Although stepwise (∼2% reduced during each phase) sucrose fading is commonly used for ethanol self-administration due to the aversion that rats initially display for ethanol taste, we followed a previously established modified sucrose fading protocol in which rats are first trained to self-administer sucrose in operant chambers, are briefly exposed to 10% sucrose and 10% ethanol for 3 days, the sucrose is then faded to 5% for one day, and the rats are subsequently moved immediately to ethanol self-administration [55]. There were no significant differences between RYGB and Sham rats during the operant shaping procedure.

Once stabilized lick responses were obtained, training on a PR10 schedule of reinforcement began. Rats were placed in the operant chambers with three spouts: Spout 1 (left – “ethanol” spout) contained one of the three ethanol concentrations, while spouts 2 (middle – “active/operant” spout) and 3 (right – “inactive” spout) were empty. Upon program activation, empty spouts 2 and 3 were presented, with licks on the inactive spout producing no programmed consequences and licks on the active spout counting towards completion of the PR10 lick contingency schedule, meaning the requirement for sucrose access increased by 10 licks per reinforcement (PR10: i.e. 10, 20, 30, etc.). If a subject did not meet the scheduled requirement after 10 minutes, the session was terminated without a reinforcement reward, providing the animals' breakpoint (operationally defined as the number of reinforcement cycles completed). Assuming the subject reached the active spout requirement, the ethanol spout was presented for a 10 s interval, during which time the licks for ethanol were recorded. At the end of the 10-s interval, the spout retracted and the procedure was repeated. Licks on all three spouts were measured. Three concentrations of alcohol (2, 4, and 8%) were tested in increasing order by concentration, and each concentration was tested for 3 consecutive days for a total of nine days on the PR10 ethanol schedule.

Following PR testing for alcohol concentrations, the rats were injected intraperitoneally (IP) with the GHS-R1A antagonist D-[Lys3]-GHRP-6 (50, 100 nmol/kg) or vehicle (sterile isotonic saline) 30 minute before initiation of a PR10 session. These doses were chosen based on previous studies demonstrating the efficacy of this drug to reduce alcohol preference and consumption in rodents [56]. A within-subject design was used where the same rats were tested each day, first with vehicle (3 days), then with the low and high dose of D-[Lys3]-GHRP-6 (once each), separated by 4 days with vehicle injections to maintain baseline and record delayed effects from drug injections. Following the second (high dose) of D-[Lys3]-GHRP-6 injection, the rats received two additional test days with vehicle injections.

### Data Analysis

Body weight (g) and food intake (kcals) were measured daily and are presented as Mean ± SEM of Group (RYGB or Sham) and were analyzed using ANOVA with Group (RYGB or Sham) and ethanol Concentration (2%, 4% or 8% EtOH) as the independent factors.

During phase one of the experiment, responses for EtOH concentrations were measured and are presented as Mean ± SEM of Group (RYGB or Sham). The number of licks made on the active, inactive, and EtOH spout, as well as the number of cycles completed, were measured and analyzed as dependent factors using two-way factorial ANOVAs with Group (RYGB or Sham) and Concentration (2%, 4% or 8% EtOH) as the independent factors.

During phase two of the experiment, the responses to 4% EtOH were measured following vehicle (saline) or a Ghrelin receptor antagonist (D-[Lys3]-GHRP-6). The number of licks made on the active, inactive, and EtOH spout, as well as the number of cycles completed were analyzed as dependent factors using two-way factorial ANOVAs with Group (RYGB or Sham) and Drug (vehicle, 50 nmol or 100 nmol D-[Lys3]-GHRP-6) as the independent factors. Data are presented as Mean ± SEM of Group (RYGB or Sham). All significant findings were further analyzed using Fisher's LSD post hoc testing. All analyses were conducted using Statistica 8.0 (StatSoft, Inc; Tulsa OK).

## Results

### Body Weight and Food Intake

Preoperative body mass for RYGB and Sham was 741.53±8.47 g, and 729.77±12.35 g, respectively, and groups did not significantly differ. All rats lost weight uniformly by the end of the first post op week (RYGB: 660.75±10.34 g, Sham: 678.43±16.73 g). In contrast, by the end of the 3^rd^ postoperative week RYGB rats displayed a loss of 18% from their pre-surgical weight, whereas the Sham-operated rats fully regained body weight to the preoperative level (data not shown).

### Oral Glucose Tolerance Tests

Fasting blood glucose levels and responses to intragastric glucose load were tested in each rat 3 weeks prior to the behavioral test, i.e. ∼3 months after the surgeries. Fasting blood glucose levels were normal and did not differ between groups (RYGB: 89.14±6.16 mg/dl, Sham: 96.75±4.16 mg/dl; F_1,6_ = 0.006, P = 0.940). Blood glucose concentrations were not statistically different at 30 and 60 min after glucose challenge between RYGB and Sham cohorts (30 min: 137.35±6.81 vs. 144.75±5.31, 60 min: 119.57±7.01 vs. 120.75±5.26, 90 min: 107.71±6.62 vs. 118.33±5.94, 120 min: 105.79±5.58 vs. 114.50±6.37).

During the experiment, body weight was significantly lower in RYGB rats as revealed by ANOVA. We found a significant effect of Group (F(3,51) = 20.8274, p<0.001), but not EtOH (2%, 4%, or 8%) Concentration (F(6,98) = 1.6339, p = 0.15) nor a Group by Concentration interaction (F(6,98) = 0.4745, p = 0.83) on body weight. Post hoc testing revealed that RYGB rats had a lower body weight across all days of testing (p<0.001; Fig. 1a). Figure 1b depicts high fat diet intake by RYGB and Sham groups throughout the study, presented in kilocalories consumed across a 24 hour period. The multivariate ANOVA revealed a significant effect of Concentration (F(6,98) = 4.0388, p<0.01) but not Group (F(3,51) = 2.0012, p<0.13) nor a Group by Concentration interaction (F(6,98) = 1,4579, p = 0.20). Post hoc analysis revealed that on Day 4 (Day 1 of testing 4% EtOH), RYGB rats ate significantly more in HF diet (kcals, p<0.05; Fig. 1b).

### Experiment 1: PR10 responses to various EtOH concentrations in RYGB and Sham rats

Animals trained to work for a reward of orally self-administered EtOH were tested using three concentrations of EtOH (2%, 4%, and 8%) and responses by RYGB and Sham rats are shown in Figure 2. Figure 2a represents the average number of licks made on the EtOH spout by RYGB and Sham rats. The factorial ANOVA revealed a significant effect of Group (F(3,46) = 4.9246, p<0.01) and EtOH Concentration (F(6,92) = 1.2705, p = 0.2787) but not Group by EtOH Concentration interaction (F(6,92) = 1.6440, p = 0.1439). Post hoc analysis showed that RYGB rats made significantly more licks on the spout containing 2% EtOH (Day 1: p<0.01), 4% EtOH (Day 5: p<0.05; Day 6: p<0.01) and 8% EtOH (Day 8: p<0.05; Day 9: p<0.01; Fig. 2a).

Figure 2b represents the average number of licks made on the active spout by RYGB and Sham rats. The factorial ANOVA revealed a significant effect of Group (F(3,46) = 5.5204, p<0.01) and EtOH Concentration (F(6,92) = 3.5126, p<0.01) but not an interaction effect (F(6,92) = 0.6858, p = 0.6615). Post hoc analysis showed that RYGB rats licked significantly more on the active spout to access 2% EtOH (Day 1: p<0.01, Day 2: p<0.05), 4% EtOH (Day 6: p<0.01, Day 7: p<0.05) and 8% EtOH (Day 9: p<0.05; Fig. 2b). Post hoc tests also revealed that on the first day of exposure for each EtOH concentration, RYGB rats made more licks for 2% EtOH (Day 1) compared to 4% EtOH (Day 4, p<0.01) and 8% EtOH (Day 7, p<0.001).

The average number of completed cycles for RYGB and Sham rats is depicted in Figure 2c. ANOVA revealed a significant effect of Group (F(3,46) = 7.5465, p<0.001) and EtOH Concentration (F(6,92) = 4.4775, p<0.001) but not an interaction effect (F(6,92) = 1.0528, p = 0.3968). Post hoc tests revealed that RYGB rats completed more cycles when testing for 2% ETOH (Day 1: p<0.01), 4% ETOH (Day 4: p<0.05; Day 6: p<0.01) and 8% ETOH (Day 8: p<0.05; Day 9: p<0.01, Fig. 2c). Post hoc testing also revealed that both groups completed more cycles for 2% EtOH (Day 1), but only on the first day of exposure. Both RYGB and Sham rats completed less cycles when working for 4% EtOH (Day 4, RYGB: p<0.01; Sham: p<0.05) and 8% EtOH (Day 7, RYGB: p<0.001; Sham: p<0.05).

For the number of licks made on the inactive spout (for which there were no programmed consequences or rewards; Fig. 2d), ANOVA revealed no significant effect of Group (F(3,46) = 1.3549, p = 0.2684), EtOH Concentration (F(6,92) = 1.9012, p = 0.0889) or interaction effect (F(6,92) = 0.2872, p = 0.9417).

### Experiment 2: Effects of D-[Lys3]-GHRP-6 on PR10 responses for EtOH

In the second study, animals were injected with vehicle or one of two concentrations of the Ghrelin receptor antagonist D-[Lys3]-GHRP-6. Figure 3a depicts the number of licks made on the reward spout containing 4% EtOH following injection of vehicle (saline; ip on baseline and post- D-[Lys3]-GHRP-6 days) or D-[Lys3]-GHRP-6 (50 nmol or 100 nmol; ip). The factorial ANOVA revealed a significant effect on EtOH licks made by RYGB and Sham rats (F(1,119) = 40.410, p<0.001) and an effect of Drug (F(6,119) = 2.3402, p<0.05) but not a Group by Drug interaction (F(6,119) = 0.5190, p = 0.7930). Compared to vehicle, D-[Lys3]-GHRP-6 did not significantly alter EtOH licks on the day that it was given for either dose tested in both RYGB and Sham rats. However, it reduced the number of EtOH licks made by RYGB rats the day after administration of 100 nmol (Post-D1-100: p<0.05) and two days after administration of 50 nmol (Post-D2-50: p<0.05), indicating a possible delayed, carry over effect of the Ghrelin receptor antagonist. Post hoc testing showed that RYGB rats made significantly more licks for EtOH on all but the day immediately after D-[Lys3]-GHRP-6 injections (Vehicle: p<0.001; 50 nmol: p<0.01; Post D1-50: p = 0.07, Post D2-50: p<0.05; 100 nmol: p<0.05; Post D1-100: p = 0.10; Post D2-100: p<0.01).

D-[Lys3]-GHRP-6 also reduced the number of licks made on the active spout towards the completion of the requirement for an EtOH reward (Figure 3b). The factorial ANOVA revealed a significant effect of Group (F(1,119) = 27.5315, p<0.001) and Drug (F(6,119) = 2.1824, p<0.05) but not a Group by Drug interaction (F(6,119) = 1.5942, p = 0.1547). Post hoc tests revealed that D-[Lys3]-GHRP-6 reduced the number of licks made by RYGB rats on the active spout following 100 nmol (p<0.05) and on the days following both doses (Post D1-50: p<0.05; Post D2-50: p<0.01; Post D1-100: p<0.05). Overall, RYGB rats made more licks on the active spout compared to Sham rats after injection of vehicle (p<0.001) and 50 nmol (p<0.001) and again on the last day of testing (Post D2-100: p<0.01).

Figure 3c shows the average number of completed cycles by RYGB and Sham rats following peripheral injection of D-[Lys3]-GHRP-6 or vehicle. The factorial ANOVA again revealed significant effect of Group (F(1,119) = 38.1615, p<0.001) and Drug (F(6,119) = 2.9284, p<0.01) but not a Group by Drug interaction (F(6,119) = 0.5650, p = 0.7574). Post hoc analysis showed that although D-[Lys3]-GHRP-6did not alter the number of cycles completed on the day of injection, there again appeared to be a carry-over effect on days following the injections (Post D2-50: p<0.05; Post D1-100: p<0.05). We also found that RYGB rats completed more cycles on several days independent of Drug (Vehicle: p<0.01; 50 nmol: p<0.01; 100 nmol: p<0.05 and Post D2-100: p<0.01).

Table 1 shows the number of licks made on the inactive spout on each day of D-[Lys3]-GHRP-6 testing. The factorial ANOVA found no significant effect of Group (F(1,119) = 3.3284, p = 0.071), Drug (F(6,119) = 1.1026, p = 0.3639) or Group by Drug interaction (F(6,119) = 0.3313, p = 0.9193), indicating that the reductions in EtOH licks and completed cycles on the post-D-[Lys3]-GHRP-6 were not due to general inactivity.

## Discussion

To our knowledge, the data presented here represent the first direct assessment of incentive motivation and consumption of ethanol following the increasingly popular Roux-en-Y gastric bypass procedure in an outbred dietary obese rat strain. Although our rats did not differ in preoperative body weight, significant differences were observed after postoperative week three, and continued throughout the course of our experiments. As can be seen in Fig. 1a, RYGB rats had significantly lower body weights than their Sham counterparts throughout the 20 experimental days, indicative of successful surgical results and corroborated by other RYGB studies that consistently show decreased body weight following RYGB surgery [5], [8], [10], [27], [36]. Although our rats did display the characteristic changes in body weight, these were not accompanied by significantly increased high fat diet consumption during testing, as our subjects only showed significantly increased diet consumption on one of the 20 test days (Fig. 1b).

Perhaps of more interest, we found that RYGB rats displayed significantly increased drug-seeking behavior when compared to their Sham controls. As can be seen from Fig. 2b, rats that underwent RYGB made significantly more responses on the active spout to earn an alcohol reward during the majority of the testing sessions for 2, 4, and 8% ethanol. Accordingly, subjects that underwent RYGB also had significantly higher breakpoints (cycles completed) than their Sham controls during the majority of the test sessions, and particularly for 4 and 8% ethanol, as can be seen in Figure 2c. Increased active spout responding and higher breakpoints denote that RYGB subjects were more willing to work for ethanol reward than controls, indicative of increased incentive motivation or “wanting” of the drug. Thus, although the literature suggests that RYGB may reduce the incentive (rewarding) effects elicited by certain foods, the converse may be true with regard to alcohol. Our current data corroborate recent clinical reports that show increased susceptibility to alcohol abuse following gastric bypass surgery [14], [15], [16], [19] and are consistent with observation of augmented alcohol preference in dietary obese rats that received RYGB [27].

Also of interest was that our RYGB subjects showed significantly increased consummatory behavior when compared to Sham controls. As can be seen from Fig. 2a, RYGB rats made more licks on the ethanol spout than Sham control subjects. Although this is an operationally defined measure of intake, the results show that particularly once acclimated to the behavioral paradigm, RYGB rats made significantly more lick-responses for 4% and 8% ethanol on the last five test days. It could be suggested that the augmented intakes are due to increased caloric need. However, the rats consume such little ethanol during the 30 minute behavioral sessions due to the high PR requirement, which makes it highly unlikely that caloric need is the decisive factor. Indeed, recent clinical studies found no correlation between increased alcohol use and the degree of weight loss [17]. Moreover, distinct to RYGB patients there were no changes in alcohol use reported in patients that underwent a laparoscopic adjustable gastric band [16], which indicates that increased alcohol intake is not a compensation for restricted meal-size. Rather, these observations of increased responding for ethanol reward, and increased consummatory behavior are more likely a result of changes in incentive motivation which is mediated through the DA reward system and modulated by peripheral hormones involved in the gut-brain system.

We next measured the responses of RYGB and Sham rats to work for and consume 4% EtOH following intraperitoneal injection of the GHS-R1A antagonist D-[Lys3]-GHRP-6. Rats working for 4% EtOH on a PR-10 schedule of reinforcement did not reduce intake immediately following either dose of D-[Lys3]-GHRP-6 (Fig. 3a), but RYGB rats did reduce the number of licks made on the active spout following 100 nmol D-[Lys3]-GHRP-6 whereas the same dose was ineffective on the Sham rats (Fig. 3b). Furthermore, for all behavioral measures RYGB rats showed significant reductions in the days following D-[Lys3]-GHRP-6 injections (Figs. 3a, 3b, and 3c). This delayed response could be due to a carry-over effect from the D-[Lys3]-GHRP-6 injections (for the higher dose), and may also indicate that the ghrelin system is involved in long-term (associative and learning) aspects of ethanol-seeking and consumption. Furthermore, these data posit that RYGB rats may be more sensitive to inactivation of the ghrelin system, strongly indicating a change in ghrelin sensitivity following RYGB surgery.

While our observation of increased alcohol reward after RYGB is supported by various human studies, several factors may explain the differential results seen here from those in a prior rodent study [26]. One likely factor is the selectively bred rat strain used in the Davis et al. study, which is quite different than the diet induced obese Sprague Dawley rats used in the current investigation, and therefore, differential results could be expected. Another factor could be differences in procedures between these two studies, i.e. no sucrose fading was used in the Davis et al. investigation. Although EtOH preferring rats seem more likely to readily consume ethanol, it is known to be aversive to rats, which is why some form of sucrose fading is used in most EtOH ingestion paradigms involving rodents. Additional differences in the paradigms include the use of a two bottle test as a measure of ethanol intake, while CPP was used as a measure of reward. Thus, strain and procedural differences are likely to underlie the discrepancies between these two studies.

RYGB has gained popularity due to its ability to produce long-lasting changes such as reduced appetite, decreased body weight, improved glucose control, and changes in taste preference [57], [58]. Despite its increasing popularity, the precise underlying mechanisms through which RYBG may produce these changes remain to be determined. Reports indicate that a number of gastrointestinal hormones change following surgery, including the orexigenic hormone ghrelin [59], [60]. Since recent studies in rodents showed that ghrelin increased their ethanol consumption and ghrelin antagonism blocked alcohol's rewarding effects [43], [50], we investigated how ghrelin influenced EtOH intake in our surgical model.

Ghrelin may influence both food intake and the motivation to obtain palatable foods and rewarding substances through its actions in the ventral tegmental area (VTA) where the cell bodies of the dopamine reward system are located [61], [62], [63], [64], [65], [66]. Ghrelin has been shown to stimulate dopamine neurons and to increase dopamine release in terminal areas of the mesolimbic dopamine system, including the nucleus accumbens [67], [68], [69]. Indeed in obese subjects a recent PET study reported that ghrelin levels were inversely associated with D2R availability in limbic brain regions including ventral striatum (location of the nucleus accumbens), such that higher levels were associated with lower D2R availability presumably from increased dopamine release and receptor occupancy [41]. Because of the multiple factors that modulate the activity of dopamine neurons, it is remarkable that ghrelin exerts such a strong stimulatory influence on alcohol reward as evidenced by the lack of alcohol-induced dopamine increases in ghrelin knock-out mice [70]. Our finding that RYGB rats were more sensitive to D-[Lys3]-GHRP-6 in reducing their alcohol intake compared to controls suggests improvement in ghrelin signaling after the surgery. Irrespective of whether such improvements affect peripheral or central ghrelin signaling or both, it may also influence the neuronal and hormonal signals upstream of the reward system. Should this effect (i.e. increased alcohol reward) rely on the dopamine system, then it may be that RYGB can improve the sensitivity of the dopamine system, which is believed to be blunted in obesity [71], [72], [73]. In fact, gastric bypass has been shown to increase the expression of dopamine D2 receptors in the ventral striatum and caudate nucleus, although the reports are still mixed [74]. As the ventral striatum is believed to be involved in modulating alcohol's rewarding effects, such changes could be especially impactful on alcohol intake and abuse potential [30].

In summary, we show that RYGB rats increased alcohol reward and consumption of low EtOH concentrations compared to their dietary obese controls, which supports the clinical findings that bariatric surgery is associated with an increased risk for alcohol abuse. We also found that changes in the responsiveness of the ghrelin system may be partly involved in the modulation of alcohol intake following RYGB, although further studies are needed to determine the precise role of ghrelin on these responses.
